# Neuroanatomical Correlates of Theory of Mind Deficit in Parkinson’s Disease: A Multimodal Imaging Study

**DOI:** 10.1371/journal.pone.0142234

**Published:** 2015-11-11

**Authors:** María Díez-Cirarda, Natalia Ojeda, Javier Peña, Alberto Cabrera-Zubizarreta, María Ángeles Gómez-Beldarrain, Juan Carlos Gómez-Esteban, Naroa Ibarretxe-Bilbao

**Affiliations:** 1 Department of Methods and Experimental Psychology, Faculty of Psychology and Education, University of Deusto, Bilbao, Basque Country, Spain; 2 OSATEK, MR Unit, Hospital of Galdakao, Galdakao, Basque Country, Spain; 3 Neurology Service, Hospital of Galdakao, Galdakao, Basque Country, Spain; 4 Neurodegenerative Unit, Biocruces Research Institute; Neurology Service, Cruces University Hospital, Baracaldo, Biscay, Spain; Bellvitge Biomedical Research Institute-IDIBELL, SPAIN

## Abstract

**Background:**

Parkinson’s disease (PD) patients show theory of mind (ToM) deficit since the early stages of the disease, and this deficit has been associated with working memory, executive functions and quality of life impairment. To date, neuroanatomical correlates of ToM have not been assessed with magnetic resonance imaging in PD. The main objective of this study was to assess cerebral correlates of ToM deficit in PD. The second objective was to explore the relationships between ToM, working memory and executive functions, and to analyse the neural correlates of ToM, controlling for both working memory and executive functions.

**Methods:**

Thirty-seven PD patients (Hoehn and Yahr median = 2.0) and 15 healthy controls underwent a neuropsychological assessment and magnetic resonance images in a 3T-scanner were acquired. T1-weighted images were analysed with voxel-based morphometry, and white matter integrity and diffusivity measures were obtained from diffusion weighted images and analysed using tract-based spatial statistics.

**Results:**

PD patients showed impairments in ToM, working memory and executive functions; grey matter loss and white matter reduction compared to healthy controls. Grey matter volume decrease in the precentral and postcentral gyrus, middle and inferior frontal gyrus correlated with ToM deficit in PD. White matter in the superior longitudinal fasciculus (adjacent to the parietal lobe) and white matter adjacent to the frontal lobe correlated with ToM impairment in PD. After controlling for executive functions, the relationship between ToM deficit and white matter remained significant for white matter areas adjacent to the precuneus and the parietal lobe.

**Conclusions:**

Findings reinforce the existence of ToM impairment from the early Hoehn and Yahr stages in PD, and the findings suggest associations with white matter and grey matter volume decrease. This study contributes to better understand ToM deficit and its neural correlates in PD, which is a basic skill for development of healthy social relationships.

## Introduction

Parkinson’s disease (PD) is a neurodegenerative disease comprising both motor symptoms and cognitive impairment [1]. Recent studies suggest that PD patients also suffer from deficits in social cognitive abilities, such as recognition of emotional prosody [2], facial emotion recognition [2–4], decision-making [4], irony comprehension [5] and specifically, Theory of Mind (ToM) deficit [3,5–7] from the early stages of the disease [3,7].

ToM was described as the ability to make inferences about others’ mental states for the first time by Premark and Woodruff [8]. More recently, ToM has been characterised as the ability to understand and predict another’s beliefs, intentions, emotions, behaviour and knowledge [6,7]. Scientific interest in ToM deficit and its cerebral correlates in PD is increasing. It has been suggested that social cognitive impairment may appear before motor symptoms in PD [3], and that ToM may play a relevant role in the dementing process [9,10]. Moreover, ToM deficit has been associated with impairment in PD patient’s quality of life [6,7,10]. ToM performance has been related to working memory and executive functions in healthy subjects [11]. The few studies that have assessed this association in PD [5,12], have reported that executive functions and working memory are involved in ToM, enhancing its performance. However, contradicting results have also been reported, suggesting the need to further explore this relationship [10,13].

ToM is related to the mirror-neuron system, whose core regions are located in the rostral part of the inferior parietal lobe, the precentral gyrus and the inferior frontal gyrus [14]. Magnetic Resonance imaging (MRI) studies have described a core network for ToM that includes the medial prefrontal cortex, bilateral posterior temporo-parietal junction [15,16] and the superior temporal sulcus [15]. Other regions, such as the precuneus and the anterior cingulate cortex, are also associated with ToM performance [15]. These findings are supported by common findings in non-human primates [14–16], autism disorder [15,16], lesion studies [16], schizophrenia [16] and healthy adults [14–16]. These anatomical areas related to ToM are known to be progressively impaired in PD [17,18]. However, to date, as far as the authors are aware, there are no studies in PD assessing the neuroanatomical correlates of ToM deficit using MRI.

The main objective of this study was to assess grey matter (GM) and white matter (WM) correlates of ToM deficit in PD. The second objective was to explore the relationships between ToM, working memory and executive functions, and to analyse the cerebral correlates of ToM, after controlling for these two cognitive functions. Voxel-based morphometry (VBM) and tract-based spatial statistics (TBSS) were used to analyse the neural correlates of ToM deficit in PD. Among diffusion tensor imaging (DTI) indexes, fractional anisotropy (FA) is the most frequently evaluated and has been related to fibres integrity, mean diffusivity (MD) has been related inversely to membrane density, radial diffusivity (RD) has shown associations with demyelination and axial diffusivity (AD) has shown increment with brain maturation but also decrement in axonal injury [19].

We hypothesised that ToM deficit in PD would correlate with GM volume and WM in the medial prefrontal cortex, temporo-parietal junction and superior temporal sulcus, all core regions related to ToM. Finally, we hypothesised that ToM performance in PD patients would correlate with executive functions and working memory. Working memory and executive functions are related to the frontal lobes [13,20,21] hence, we hypothesised that the influence of executive functions and working memory on ToM would be reflected in the medial prefrontal cortex, reducing the association between the frontal areas and ToM.

## Materials and Methods

### Subjects

The sample included 44 PD patients recruited from the Department of Neurology at the Galdakao Hospital and from the PD Biscay Association (ASPARBI). The main purpose of the study was to analyze the neuroanatomical correlates of ToM deficit in PD; however, we also recruited 15 healthy controls, who were matched with the patients by age, gender and years of education, to explore differences between groups.

PD patients were enrolled in the study if they fulfilled the UK PD Society Brain Bank diagnostic criteria. Other inclusion criteria were as follows: i) age between 45–75; ii) Hoehn and Yahr disease stage < 3 [22]; iii) Unified PD Rating Scale (UPDRS) [23] evaluated by the neurologist. The exclusion criteria were as follows: i) the presence of dementia as defined by the DSM-IV-R [24] and the Movement Disorders Society clinical criteria for PD-dementia; ii) scores on the Mini Mental State Examination < 24; iii) the presence of other neurological illness/injury (e.g. traumatic brain injury); iv) unstable psychiatric disorders (e.g. schizophrenia); v) PD patients with visual hallucinations as assessed by the Neuropsychiatric Inventory Questionnaire [25]; and vi) Diagnosis of depression or depression evaluated with the Geriatric Depression Scale >5 [26]. WM hyperintensity ratings were calculated twice by the same neuroradiologist using the Fazekas Scale [27] based on T1-weighted images. Considering that some degree of WM hyperintensity is typical in the elderly, these criteria did not exclude any of the participants. Five patients were excluded due to exclusion criteria and 2 patients refused to participate, therefore the final PD sample consisted of 37 PD patients.

One patient was taking no medication and 36 were on anti-Parkinsonian treatment as follows: Levodopa (L-dopa) monotherapy (n = 4), combination of L-dopa and dopamine agonist (n = 5), monoamine oxidase type B (MAO-B) inhibitors monotherapy (n = 1), combination of L-dopa and MAO-B (n = 5), combination of L-dopa, dopamine agonist and MAO-B (n = 9), combination of dopamine agonist and MAO-B (n = 4), combination of dopamine agonist and anticholinergics (n = 2), combination of glutamate agonists in combination with others (n = 4), catechol-O-methyltransferase (COMT) inhibitors in combination with others (n = 2). Participants were symptomatically stable and tested while on their medication. Their L-dopa equivalent daily dose was registered [28].

### Ethics Statement

The study protocol was approved by the Ethics Committee at the Health Department of the Basque Mental Health System in Spain. All subjects were volunteers and provided written informed consent prior to their participation in the study, in accordance with the Declaration of Helsinki.

### Neuropsychological assessment

Participants underwent a neuropsychological battery including the Spanish version of the Mini-Mental State Examination [29] as a screening cognitive measure. Digit Span Backward [30] was used to assess working memory, and the Clock Drawing Test (order) [31] and Verbal fluency Test (phonetic and semantic) [32] to evaluate executive functions. Executive functions and working memory tests were chosen following the recommendations by the Movement Disorders Society Task Force for diagnosis of mild cognitive impairment in PD [33]. ToM was assessed with the Spanish version of the Happe Test “Strange Stories Task” [34], developed by Pousa [34,35] (an Advanced Test of Theory of Mind), and the global score was selected for correlation with diffusion weighted images and T1 derived measures. Happe test is composed of 8 stories concerning double bluff, mistakes, persuasion and white lies, and has been previously used for the assessment of the neuroanatomical correlates of ToM [34,36]. This study is part of a more extensive longitudinal study; therefore, to avoid test-retest learning effect at longitudinal evaluation, we evaluated ToM with 4 stories at first time (included in this study) and 4 at second time. Participants had to read aloud each story and then, answer a question requiring an inference about the character’s thoughts, which required an inference about the speaker’s/actor’s intentions. The participant was asked to answer the required questions, explaining his/her point of view, after demonstrating that he/she understood the task with an example story. Responses were scored between 0 and 2, strictly following the instructions of the manual, where explicit answers were scored with 2 points and implicit answers with 1 point and no response or non-related responses with 0 points. A trained neuropsychologist gave the score of the Happe Task to each participant, guided by the definition and examples showed in the manual to score explicit and implicit answers. Results of ToM performance in this PD sample have been previously published in a longitudinal study [37], and results showed that PD patients, who received cognitive rehabilitation therapy, improved their ToM performance.

### Image acquisition and analysis

Diffusion-weighted images were obtained on a Phillips 3T Achieva, in an axial orientation in an anterior-posterior phase direction using a single-shot EPI sequence (TR = 7540 and TE = 76, matrix size = 120mm x 117mm; flip angle = 90°, FOV = 240x240x132, slice thickness = 2 mm, no gap, 66 slices, acquisition time = 9’31”, voxel size = 1.67x1.67x2.0) with diffusion weighting in 32 uniformly distributed directions (b = 1,000 s/mm^2^) and 1 b = 0 s/mm^2^. A T1-weighted scan was also acquired in sagittal orientation (TR = 7.4 and TE = 3.4, matrix size = 228mm x 218mm; flip angle = 9°, FOV = 250x250x180, slice thickness = 1.1 mm, 300 slices, acquisition time = 4’55”, voxel size = 0.98x0.98x0.6).

#### VBM analysis in GM

VBM analyses were carried out using the FMRIB Software Library (FSL) [38] tools (http://fsl.fmrib.ox.ac.uk/fsl/fslwiki/FSLVBM) [39]. First, a study-specific template was created so that all images could be registered in the same stereotactic space (spatial normalisation). To do this, brain-extracted structural images were segmented into GM, WM, and cerebrospinal fluid. Then, GM images were affine registered to the GM ICBM-152 template and averaged to create an affine GM template. Next, GM images were re-registered to this affine GM template using a non-linear registration and averaged to create the study-specific non-linear GM template in standard space. Second, individual GM images were registered non-linearly to the study-specific template. After the normalisation, the resulting GM images were modulated by multiplying with Jacobian determinants to correct for the volume change induced by the nonlinear spatial normalisation. Finally, the images were smoothed with sigma of 3.5 mm (8 mm FWHM).

#### TBSS analysis in WM

Diffusion data were preprocessed and analysed using tools from FSL [38]. First, each subject’s images were concatenated and radiologically oriented. Then, the data were corrected for motion and eddy currents, performed brain-extraction BET, and the diffusion gradients (bvecs) were rotated to be corrected accordingly, providing a more accurate estimate of tensor orientations [40]. Then, all FA, MD, RD and AD images were obtained by fitting a tensor model to the raw diffusion data using FDT (DTIFIT). After, TBSS [41] was used for group comparisons and correlations analyses. Using TBSS, the data were prepared to apply a nonlinear registration of all FA images into standard space, the mean FA image was created using a threshold of 0.2 and thinned to create a “mean FA skeleton” which represents the centres of all tracts common to the group. MD data were analysed using “tbss non FA” script from TBSS, which applies the original non lineal registration to the MD data, merges all subjects warped MD data into a 4D file, then project this onto the original mean FA skeleton, and creates the 4D projected data. The same process was repeated for RD and AD.

### Statistical analysis

All variables were tested for normality. Differences between groups were assessed with t-test, chi-squared (*X* ^2^) test and ANOVA for neuropsychological differences. Correlation analysis was performed with r-Pearson. Raw scores were transformed into Z scores. Executive functions were measured using a composite score, calculated from phonetic and semantic Verbal Fluency Test and the Clock Drawing Test (order) (alpha = .747). In addition, test-retest reliability for Fazekas Scale was performed and correlation analysis calculated (Spearman´s Rho = .835; *p* < .001). Age and gender were introduced as nuisance variables in neuropsychological and neuroimaging analyses.

Whole-brain VBM differences between groups and the relationships between GM volume and ToM were analysed with randomise tool [42] (5000 permutations) and with threshold-free cluster enhancement (TFCE). Total intracranial volume was calculated, transformed to a Z score and introduced as a covariate in between-group analysis, following previous GM studies [43–45]. The significant regions were located and labelled anatomically with the Harvard-Oxford Cortical Structures Atlas. Statistical threshold for VBM analysis was set at *p* < .05 corrected for multiple comparisons using family wise error (FWE). Exploratory analyses were also performed at *p* < .001 (uncorrected) level, with minimum extended cluster K>20 voxels to be considered as a significant result. To examine the differences between groups in WM FA, RD, MD and AD and to assess the relationships between WM indexes and ToM, randomise tool [42] (5000 permutations) and a regression analysis with TFCE correction was used. The significant regions were located and labelled anatomically with the JHU-ICBM-DTI-81 WM Labels and JHU White-Matter tractography Atlas. Statistical threshold was set at *p* < .05 (FWE-corrected), and exploratory analyses using *p* < .001 (uncorrected, K>20 voxels) were also reported. Effect sizes for each cluster of the group comparisons and correlations were calculated according to Cohen´s *d* formula or *r* formula respectively, in line with previous investigations [46–48]. Cohen´s *d* of 0.20, 0.50 and 0.80 were considered small, medium and large, respectively [49]. Interpretation of *r* score was considered small, medium and large when scores were 0.10, 0.30 and 0.50, respectively [49].

## Results

### Neuropsychological results

The clinical and sociodemographic characteristics of the sample are shown in Table 1.

**Table 1:** Sociodemographic, clinical and neuropsychological characteristics of the sample.

		PD (n = 37)	HC (n = 15)	Statistic	*p*
**Sociodemographic and clinical characteristics**					
Age		67.97 (6.17)	65.07 (7.01)	t = 1.47	.15
Gender (Male)		22 (59.5%)	11 (73.3%)	*X* ^2^ = .88	.52
Years of education		10.24 (4.81)	12.27 (4.30)	t = -1.41	.16
Handedness	Right handed	33 (89.2%)	15 (100%)	*X* ^2^ = 1.75	.31
	Ambidextrous*	4 (10.8%)	0%		
Fazekas Scale		.51 (.69)	.67 (.90)	*X* ^2^ = 2.89	.23
		0 = 22	0 = 9		
		1 = 11	1 = 2		
		2 = 4	2 = 4		
Geriatric Depression Scale		2.57 (2.80)	1.20 (1.37)	t = 1.79	.08
Neuropsychiatric Inventory		3.46 (4.07)	-	-	-
Side of disease onset	Right side of the body	14 (37.8%)	-	*X* ^2^ = 1.40	.24
	Left side of the body	21 (56.8%)	-		
UPDRS	Mental State	1.86 (1.47)	-	-	-
	Daily Living Activities	10.28 (6.27)	-	-	-
	Motor Exam	21.72 (10.29)	-	-	-
	Treatment complications	2.75 (2.88)	-	-	-
	Total Score	36.61 (17.27)	-	-	-
LEDD		808.59 (536.81)	-	-	-
Years of Disease Evolution		6.96 (5.61)	-	-	-
Hoehn & Yahr		1.89 (.45)	-	-	-
		1 = 5	-	-	-
		1.5 = 3	-	-	-
		2 = 26	-	-	-
		2.5 = 1	-	-	-
		3 = 2	-	-	-
**Neuropsychological assessment**					
ToM (Z score)		-.23 (1.05)	.57 (.54)	F = 5.854	.019
Working Memory (Z score)		-.30 (.79)	.63 (.77)	F = 8.911	.004
Executive Functions (Z score)		-.26 (.74)	.56 (.59)	F = 12.57	.001
Cognitive Reserve (Z score)		-.14 (1.03)	.35 (.83)	F = 1.929	.137

Values are expressed as mean (S.D) unless otherwise noted. PD = Parkinson’s disease; HC = Healthy controls; UPDRS = Unified Parkinson Disease Rating Scale; LEDD = Levodopa Equivalent Daily dose; ToM = Theory of Mind.

*Ambidextrous understood as people who were originally left handed and who learned to be right handed during childhood.

PD participants obtained significantly lower scores on ToM, working memory and executive functions compared to healthy controls (Table 1). Moreover, a significant positive correlation was found between ToM and executive functions (r = .45; *p* = < .01), but not with working memory (r = .30; *p* = .06) in PD.

### VBM analysis in GM

#### Group Comparison

**Fig 1:**
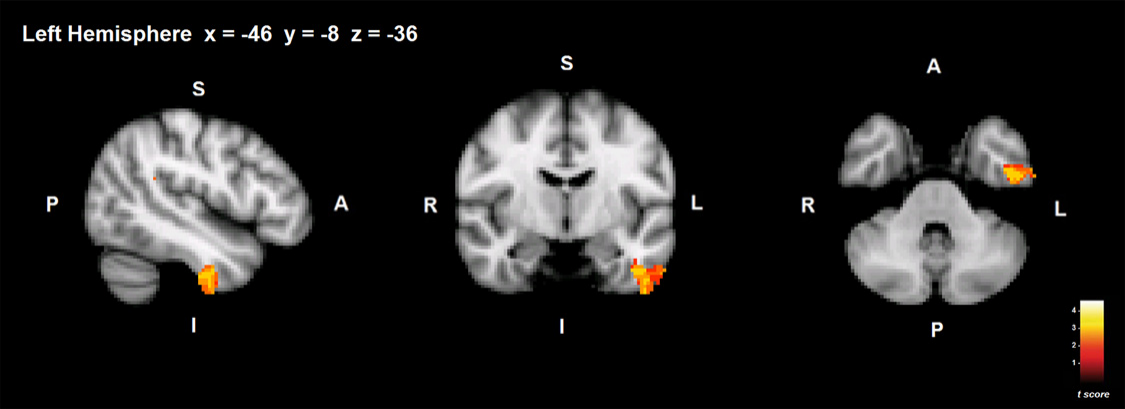
Group differences in GM volume. GM areas showing volume decrease in PD compared to healthy controls are shown in red-yellow. S = superior; I = inferior; A = anterior; P = posterior. Coordinates are shown in MNI space (Montreal Neurological Institute).

PD patients showed reduced GM volume in the left temporal, parietal and occipital lobes (*p* < .001 uncorrected). More specifically, GM regions that showed potential reductions in PD patients compared to healthy controls were mostly located in the left inferior temporal gyrus (anterior and posterior division) and the temporal fusiform cortex (Fig 1; Table 2). Other regions that also showed potential reductions were the superior and inferior parietal lobe, left supramarginal gyrus, and the lateral occipital cortex (Table 2). The healthy control group did not show any areas with reduced GM volume compared to PD patients.

**Table 2:** VBM results: Group comparison and correlation analysis with ToM in PDBrain Area.

	Cluster size (voxels)	MNI coordinate			t value	*p* value	Effect size (Cohen´s *d* / *r*) *df* = 36
		x	y	z			
**Group Comparison**							
L Inferior temporal gyrus	1973	-48	-10	-48	3.77	.001*	1.15
	267	52	-18	-40	3.05	.003*	.84
L Lateral Occipital Cortex, L Superior Parietal Lobe	745	-18	-76	56	3.33	.001*	.93
L Inferior Parietal Lobe	582	-44	-44	22	2.99	.001*	.91
R Temporal Lobe	158	44	26	-26	3.73	.003*	1.14
**Correlation with ToM in PD**							
L Precentral gyrus, L Postcentral gyrus	830	-32	-24	64	3.59	< .001*	.51
L Anterior Cingulate gyrus	147	-4	-12	28	2.24	< .001*	.34
	109	0	32	2	2.91	< .001*	.43
L Middle frontal gyrus, L Inferior frontal gyrus	84	-30	18	32	2.17	< .001*	.34

Cluster size denotes the extent of the cluster of significant voxels. MNI coordinates refer to the location of the most statistically significant voxel in the cluster.

*Differences are significant at *p* < .001 uncorrected.

PD = Parkinson’s disease; ToM = Theory of Mind; L = Left; R = Right; MNI = Montreal Neurological Institute. *df* = Degrees of Freedom.

#### Correlations between ToM and GM volume in PD

No significant correlations were observed at *p* < .05 (FWE-corrected) statistical threshold, but the exploratory analysis showed possible associations between ToM and GM volume, in the left precentral and postcentral gyrus, anterior cingulate gyrus, middle frontal gyrus and the inferior frontal gyrus in PD (Table 2) (*p <* .001 uncorrected). Healthy controls’ performance in ToM test showed no significant correlation with GM volume.

#### Correlations between ToM and GM volume in PD, controlling for executive functions

Because executive functions showed a positive association with ToM, we also included this variable as a covariate in the regression analysis. No significant clusters were obtained in the regression analysis between GM volume decrease and ToM after controlling for executive functions in PD.

### TBSS analysis in WM

#### Group Comparison

PD patients showed FA reduction in the right uncinate fasciculus adjacent to the insular cortex and slight WM FA reduction was observed in the frontal lobe compared to healthy control group (*p* < .001 uncorrected). Results showed no significant differences between PD and healthy control groups in MD, RD or AD indexes.

#### Correlations between ToM and WM in PD

**Fig 2:**
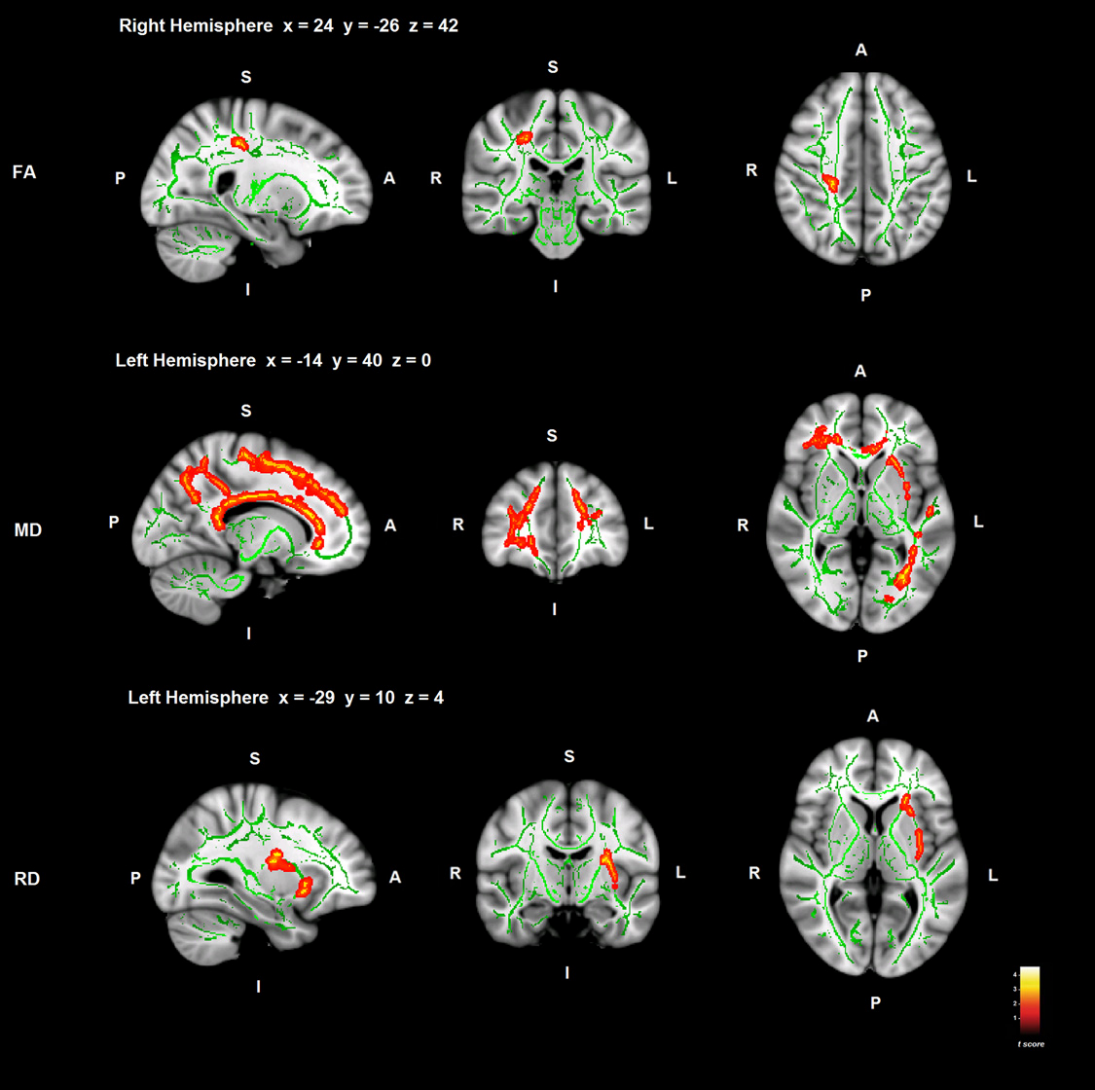
Correlations between ToM deficit and WM in PD. Significant WM regions are shown in red-yellow; the WM skeleton is shown in green. S = superior; I = inferior; A = anterior; P = posterior. Coordinates are shown in MNI space (Montreal Neurological Institute).

ToM deficit in PD patients correlated positively with FA reduction and negatively with MD and RD indexes of WM tracts. Most significant correlations were found between WM and ToM deficit in the bilateral superior longitudinal fasciculus in PD (Fig 2; Table 3).

**Table 3:** DTI results: Group comparison and correlation analysis with ToM in PD.

Brain Area	Cluster size (voxels)	MNI coordinate			t value	*p* value	Effect size (Cohen´s *d* / *r*) *df* = 36
		x	y	z			
**Group Comparison**							
**FA**							
R Uncinate Fasciculus	42	34	15	-13	2.95	.001*	.81
**Correlation with ToM in PD**							
**FA**							
R Superior Longitudinal Fasciculus	97	23	-29	42	5.18	.032**	.65
**MD**							
L Superior longitudinal Fasciculus	13046	-40	-55	36	5.86	.031**	.69
	259	-31	14	28	5.20	.040**	.65
L External Capsule	483	-33	3	4	4.14	.039**	.56
**RD**							
L Superior Longitudinal Fasciculus	650	-30	-10	14	3.8	.040**	.53
**Correlation with ToM in PD controlling for Executive Functions**							
**FA**							
R Superior Longitudinal Fasciculus	227	28	-26	43	2.61	.002*	.39
**MD**							
L Superior Longitudinal Fasciculus	1322	-40	-55	35	5.33	.049**	.66
	878	-19	-45	43	3.93	.043**	.54
L Inferior Longitudinal Fasciculus	165	-31	-69	-1	4.04	.049**	.55
**RD**							
R Corticoespinal Tract	296	14	-13	66	1.24	.002*	.20
	124	-27	-20	63	0.86	.001*	.14
L Inferior Longitudinal Fasciculus	169	-27	-63	47	1.69	.001*	.27
	115	-28	-8	-16	1.23	.001*	.20
L Superior Longitudinal Fasciculus	109	-58	-26	6	1.31	.002*	.21

Cluster size denotes the extent of the cluster of significant voxels. MNI coordinates refer to the location of the most statistically significant voxel in the cluster.

*Differences are significant at *p* < .001 uncorrected.

**Differences are significant at *p* < .05 corrected for family-wise error (FWE).

PD = Parkinson’s disease; ToM = Theory of Mind; R = Right; L = Left; FA = Fractional Anisotropy; MD = Mean Diffusivity; RD = Radial Diffusivity; MNI = Montreal Neurological Institute; *df* = Degrees of Freedom.

WM FA reduction in the right superior longitudinal fasciculus and corticospinal tract adjacent to the primary somatosensory cortex correlated with ToM deficit in PD (Brodmann Area 3a) (*p* < .05 FWE-corrected) (Fig 2; Table 3).

In addition, MD index correlated negatively with ToM deficit in PD, and significant clusters were found in the left superior longitudinal fasciculus located longitudinally in the superior frontal gyrus and premotor cortex (Brodmann Area 6), continuing through the primary somatosensory cortex in the parietal lobe, the precuneus and finishing in the occipital cortex. Furthermore, MD in the left inferior longitudinal fasciculus, the right inferior fronto-occipital fasciculus and the left uncinate fasciculus, adjacent to middle temporal gyrus, to the orbitofrontal cortex, and frontal lobe respectively also correlated with ToM deficit in PD. Finally, MD in the callosal body also correlated with ToM impairment in PD patients (Fig 2; Table 3).

Moreover, RD in the left superior longitudinal fasciculus and corticospinal tract adjacent to the secondary somatosensory cortex, in the external capsule, and in the left anterior thalamic radiation and inferior fronto-occipital fasciculus in the frontal lobe, correlated negatively with ToM deficit in PD (Fig 2; Table 3).

AD index showed no significant relationship with ToM deficit in PD. No significant correlations were found between ToM performance in healthy controls and FA, RD, MD or AD.

#### Correlations between ToM and WM in PD, controlling for executive functions

**Fig 3:**
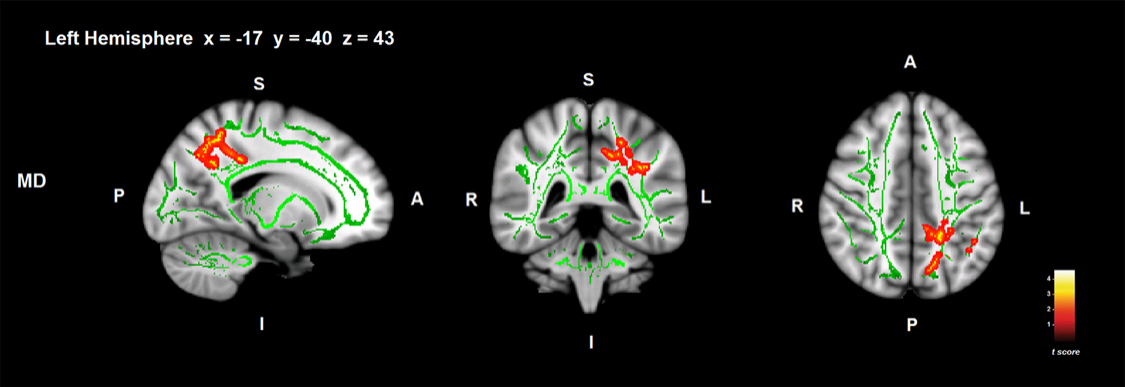
Correlations between ToM deficit and WM MD controlling for executive functions in PD. Significant WM regions are shown in red-yellow; the WM skeleton is shown in green. S = superior; I = inferior; A = anterior; P = posterior. Coordinates are shown in MNI space (Montreal Neurological Institute).

After controlling for executive functions, MD in the left superior longitudinal fasciculus adjacent to anterior intra-parietal sulcus, superior parietal lobe and precuneus showed significant associations with ToM deficit in PD (Fig 3; Table 3) (*p* < .05 FWE-corrected). Exploratory analyses showed potential associations between ToM impairment in PD and FA and RD in the right superior longitudinal fasciculus adjacent to somatosensory cortex (Table 3) (*p* < .001 uncorrected).

## Discussion

The main objective of the study was to assess the neuroanatomical correlates of ToM deficit in PD. The results suggest that ToM deficit is associated with WM in PD and possible associations with GM volume decrease. More specifically, performance on ToM task in PD was related to GM volume decrease in the left medial frontal cortex, inferior frontal gyrus, anterior cingulate gyrus and precentral gyrus, all regions known to be involved in ToM performance [14–16]. Indeed, similar results have been obtained in other neurodegenerative disorders such as progressive supranuclear palsy [43], and the anterior cingulate has also been related to ToM in Alzheimer´s disease [46]. GM volume loss in the postcentral gyrus also showed possible correlations with ToM deficit. In previous studies, the somatosensory cortex has been related to ToM deficit in autism spectrum disorder and schizophrenia [50]. Moreover, FA, MD and RD in the superior longitudinal fasciculus showed significant correlations with ToM deficit in PD. This tract connects the dorsolateral prefrontal cortex with the parieto-temporal association areas [51], involving all cortical areas related to ToM performance [16]. In addition, MD and RD in WM tracts located in the frontal lobe and specifically in the uncinate fasciculus and inferior fronto-occipital fasciculus adjacent to the orbitofrontal cortex showed significant associations with ToM deficit in PD. These findings add evidence to previous studies that related the ability to process social and emotional information to the frontal lobes in PD [4,5,13–18], and to the uncinate fasciculus and inferior fronto-occipital fasciculus in other pathologies [52,53].

However, previous studies assessing the neuroanatomical correlates of ToM in healthy controls [14–16,18], in progressive supranuclear palsy [43], autism and schizophrenia [50] did not evaluate the potential influence of working memory and executive functions in the MRI analysis. Because of contradicting results on the influence of these cognitive functions on ToM performance in PD, the second objective of the present study was to assess this relationship and to evaluate the neuroanatomical correlates of ToM deficit after controlling for both cognitive functions. In support of previous literature [5,12,13,21], a correlation between ToM and executive functions was observed in PD, but not with working memory. After controlling for the influence of executive functions, WM MD in the frontal regions that initially appeared significantly associated to ToM deficit in PD, was no longer so. The bilateral frontal cortices are also defined as neural correlates of executive functions [12,20,21]. As we hypothesised, the prefrontal and medial frontal clusters may mostly represent the influence of executive functions on ToM. The strength of the correlation between ToM deficit and WM in PD remained significant mainly in WM tracts adjacent to the parietal lobe and precuneus. The most significant cluster region in the correlation between ToM impairment and the MD of WM in PD even when controlling for executive functions, was found in the right superior longitudinal fasciculus. Studies in autism disorder concluded that WM alterations in the right superior longitudinal fasciculus [54] could be related to ToM impairment [52,54]. Despite the relationship between ToM and executive functions, both cognitive functions showed different cerebral correlates, therefore, ToM should be considered as an independent cognitive function, and ToM deficits cannot be only understood as a consequence of executive dysfunction.

One possible interpretation for the effects that are lost when executive functions are controlled may also be due to the executive functions per se, rather than to their influence on ToM. Executive functions may be part of ToM but may also be acting as a confounding variable. Furthermore, studies have demonstrated different patterns of brain volume decrease among PD patients with normal cognition, PD patients with mild cognitive impairment and demented PD patients [55,56]. Despite PD group in this study showed cognitive impairment in some cognitive functions, there may be variability in the cognitive profile among PD patients in the sample and a more extensive neuropsychological battery is needed to better characterise the cognitive status of each PD patient and the associated cerebral characteristics. Future studies should assess the different neuroanatomical correlates of ToM comparing PD patients with and without mild cognitive impairment and demented PD patients.

PD patients in this study were at relatively early Hoehn and Yahr stages of the disease, so results also reinforce the prompt appearance of ToM deficit in the disease [3,7,17]. Focusing on cerebral differences between patients and healthy controls, reduced GM volume mainly in the temporal lobe but also in the parietal and occipital lobes was found in PD patients, as reported before [44,57]. On the other hand, exploratory analysis showed slight WM differences between groups. PD patients in this study showed FA reduction in the uncinate tract, which has been related to less WM integrity [19]. In addition, PD patients showed no significant differences with healthy controls group in MD (membrane density) as suggested in previous studies [58], and neither in RD (axonal demyelination) nor AD (axonal injury) indexes. The literature suggests that WM impairment in PD occur with the progression of the disease [9,55], and that this deterioration can be detected in PD patients at moderate stages of the disease [9,55,59]. A strong correlation between ToM deficit and WM in PD was obtained. Hence, with the progression of the disease, WM differences between groups would very likely be accentuated, and the association between ToM impairment and WM disruption might be stronger. Further studies are needed to assess WM integrity and diffusivity in PD patients at both primary and later stages of the disease.

No significant correlations were found between ToM and GM or WM in heathy control group. We relate the absence of correlation between ToM performance and brain characteristics in healthy adults to the small sample size, the ceiling effect of Happe test found in our sample and consequently, the reduced variability of the data. Firstly, the small sample size makes more difficult to reach the significant level in statistical analysis. Secondly, answers in Happe test are scored between 0 and 2, and most of the healthy participants of the sample scored 7 points (maximum punctuation of 8), producing a ceiling effect. These two facts bring a reduced variability of the data. The reduced variability of the data, added to the preservation of the cerebral characteristics in healthy control group, may be the reasons for not findings significant relationships between ToM performance and GM and WM.

An important issue to consider is that GM correlates of ToM in PD and WM differences between groups were reported with uncorrected results. However, we calculated the effect sizes (Cohen’s *d* and r) of the results to support these findings. Uncorrected results reflected medium-large effect sizes which brings a useful indicator of the clinical importance of these results [49]. Moreover, the study included a minimum extended cluster K>20 voxels in neuroimaging analyses to consider results as significant findings, reducing the probability of reporting false positives in the results. Another limitation of the study is that executive functions were assessed with Verbal Fluency Test and Clock Drawing Test (order), two cognitive tests that measure executive functions in different ways, however, another test would be more representative for assessing this cognitive function, such as the Wisconsin card sorting Test (WCST) or the Tower of London Test. Despite this limitation, both tests showed a high internal consistency (Cronbach´s alpha = .747). Finally, ToM has also been related to clinical symptoms such as depression and quality of life in PD patients, future studies should also assess this relationship and test the possible moderation effects of depression in the relationship between ToM and cerebral correlates.

The lack of differences between groups in WM lesions, assessed with the Fazekas scale [27], allows us to attribute WM dysfunction in PD to the neurodegenerative process and not to vascular risk. Other studies [9,57] emphasise the use of WM hyperintensities as a covariate if differences existed between groups, to report more accurate results. However, we measured WM lesions using a T1-weighted sequence.

To summarise, the present study reinforces the presence of ToM impairment from the early Hoehn and Yahr stages of PD, and the findings suggest associations with WM integrity and with GM volume. Specifically, GM volumes in the prefrontal cortex, precentral gyrus and somatosensory cortex showed potential relationships with ToM deficit in PD. In addition, the WM in the right superior longitudinal fasciculus and corticospinal tract (adjacent to the parietal lobe), and WM tracts adjacent to the orbitofrontal cortex were related to ToM deficit in PD. However, after controlling for executive functions in the regression analysis, the associations of prefrontal regions with ToM deficit were no longer significant. This may suggest that the frontal component of ToM is due to the influence of executive functions and that “pure ToM” is related to the precuneus and parietal lobe.

Preserved ToM performance is essential for developing healthy social relationships and it is thought to have an impact on a patient´s quality of life. The study of ToM deficit in PD and its cerebral correlates increases our knowledge and may help in identifying more effective treatments to rehabilitate this function. Future studies with larger samples are needed to deeply explore the neuroanatomical correlates of ToM deficit in PD and the mediating effects of executive functions and working memory on ToM performance.
