# Correction: Neuroanatomical Correlates of Theory of Mind Deficit in Parkinson's Disease: A Multimodal Imaging Study

**DOI:** 10.1371/journal.pone.0144482

**Published:** 2015-12-02

**Authors:** The PLOS ONE Staff

The Data Availability Statement for this paper is incorrect. The correct statement is: All data are available from Figshare at the following DOI: http://dx.doi.org/10.6084/m9.figshare.1582668.

There is an error in the second sentence of the second paragraph of the “Subjects” section of the Materials and Methods. It should read: Other inclusion criteria were as follows: i) age between 45–75; ii) Hoehn and Yahr disease stage < 3 [22]; iii) Unified PD Rating Scale (UPDRS) [23] evaluated by the neurologist.

There is an error in the heading for Table 2. Please view the correct Table 2 here.

**Table 2:** VBM results: Group comparison and correlation analysis with ToM in PD.

Brain Area	Cluster size (voxels)	MNI coordinate			t value	*p* value	Effect size**(Cohen´s *d* / *r*) *df* = 36**
		x	y	z			
**Group Comparison**							
L Inferior temporal gyrus	1973	-48	-10	-48	3.77	.001*	1.15
	267	52	-18	-40	3.05	.003*	.84
L Lateral Occipital Cortex, L Superior Parietal Lobe	745	-18	-76	56	3.33	.001*	.93
L Inferior Parietal Lobe	582	-44	-44	22	2.99	.001*	.91
R Temporal Lobe	158	44	26	-26	3.73	.003*	1.14
**Correlation with ToM in PD**							
L Precentral gyrus, L Postcentral gyrus	830	-32	-24	64	3.59	< .001*	.51
L Anterior Cingulate gyrus	147	-4	-12	28	2.24	< .001*	.34
	109	0	32	2	2.91	< .001*	.43
L Middle frontal gyrus, L Inferior frontal gyrus	84	-30	18	32	2.17	< .001*	.34

Cluster size denotes the extent of the cluster of significant voxels. MNI coordinates refer to the location of the most statistically significant voxel in the cluster.

*Differences are significant at *p* < .001 uncorrected.

PD = Parkinson’s disease; ToM = Theory of Mind; L = Left; R = Right; MNI = Montreal Neurological Institute. *df* = Degrees of Freedom.

The publisher apologizes for these errors.
